# Dentists’ perspectives, practices, and factors associated with informed consent process for fixed prosthodontic treatment: a cross-sectional study of kampala metropolitan area, Uganda

**DOI:** 10.1186/s12903-024-04380-w

**Published:** 2024-05-27

**Authors:** Barbara Ndagire, John Barugahare, Sudeshni Naidoo, Joaniter Nankabirwa, Joan Nakayaga, Charles Mugisha Rwenyonyi

**Affiliations:** 1 School of Dentistry, School of Health Sciences, College of Health Sciences, Makerere University, Kampala, Uganda; 2 Department of Philosophy, School of Liberal and Performing Arts, College of Humanities and Social Sciences, Makerere University, Kampala, Uganda; 3 Faculty of Dentistry, University of the Western Cape, Bellville, South Africa; 4 Clinical Epidemiology Unit, School of Medicine, College of Health Sciences, Makerere University, Kampala, Uganda

**Keywords:** Dentists, Fixed prosthodontic treatment, Informed consent, Perspectives, Practices, Uganda

## Abstract

**Background:**

Dentists have a legal and ethical obligation to obtain informed consent from patients before carrying out treatment. In Uganda, the process of obtaining informed consent in dentistry is not well documented. The aim of the present study was to determine dentists’ perspectives and practices regarding informed consent to fixed prosthodontic treatment (FPT) in Kampala Metropolitan, Uganda.

**Methods:**

A quantitative cross-sectional study was conducted among 153 dentists from July to September 2023. Data were collected using a semi-structured self-administered questionnaire that included both closed- and open-ended questions. The questionnaire included items on participants’ sociodemographic information, perspectives, and practices about informed consent for FPT. Perspectives were rated using ten items on a five-point Likert scale. The minimum possible total score was 10, and the maximum possible score was 50. Descriptive statistics and Poisson regression were used to summarize and analyze the quantitative data, and the significance level was set at *p* < 0.05. Open-ended items were analyzed using content analysis.

**Results:**

The majority (83.9%) of the participants were general dentists with working experience ranging from 1 to 38 years and a median of 8 years. The majority were familiar with the concept of informed consent and had positive perspectives regarding its use for FPT. The mean score for perspectives was 39.27 (SD, 5.42). However, there were variations in the practices of the dentists. More than three-quarters (87.6%) reported that they always obtained the patient’s informed consent before FPT. Less than a third (29.4%) obtained written consent for FPT. About half of the dentists provided information regarding the procedure, benefits, and risks of treatment during the consent process. Bivariate analysis showed that the use of written consent for FPT was significantly (*p* < 0.05) associated with having a work experience of more than 10 years and having had training involving informed consent after undergraduate studies.

**Conclusion:**

The present study provides baseline data regarding perspectives and practices regarding informed consent for FPT among dentists in Uganda. It is recommended that regular training courses be developed to highlight the importance of improved informed consent practices for patient protection and to instruct dentists about obtaining valid informed consent. There is a need for future research to streamline guidelines for the informed consent process in dental care in Uganda.

**Supplementary Information:**

The online version contains supplementary material available at 10.1186/s12903-024-04380-w.

## Introduction

The process of informed consent is both an ethical and legal requirement in dental practice [1–3]. Informed consent is fundamental to the care, treatment, and management of dental patients [2, 4]. Informed consent has been defined as the process of communication between a patient and healthcare provider that results in the patient’s authorization or agreement to undergo a specific medical intervention [5]. Failure to obtain informed consent compromises patient autonomy places patient safety at risk, and legally, may constitute negligence or battery [3, 5].

Over the last decades, dental practice lawsuits increased when patients were unhappy with the treatment provided, and they felt that treatment options were not properly discussed [6]. In several countries the prosthodontic specialty is among the most implicated dental procedures, accounting for upto 54.5% of dental claims or litigations [7–13]. In many such claims, informed consent may be absent for instance Alsaed and colleagues found that only 10% of the dental malpractice lawsuits investigated had a consent form signed by the patients before treatment [14]. These legal issues in dentistry, for instance in prosthodontics could be avoided if routine ethical practice including informed consent was obtained [6, 15].

The World Medical Association outlines that informed consent is the primary paradigm for protecting patients’ legal rights and guiding the ethical practice of medicine [16]. In Uganda, informed consent is a legal requirement as stated in Sect. 7 of the code of professional ethics published in 2013 by the Uganda Medical and Dental Practitioners Council: “A practitioner shall not conduct any intervention or treatment without consent” [17]. Furthermore, according to Article 10 of the 2019 Patient’s Rights and Responsibilities Charter: “Every patient has the right to be given adequate and accurate information about the nature of one’s illness, diagnostic procedures, and the proposed treatment for one to make an informed decision” [18].

Available literature regarding dentists’ knowledge and practices about informed consent reveals contradictory results from different countries [19–21]. While the idea of informed consent is well understood and practiced among dentists in a few countries, there are gross deficiencies in several developing countries [21–24]. Several studies report substantial (56–55%) percentages of dentists who obtain consent only in special cases or sometimes [23, 25], or not at all [23]. In addition, there are gross variations in the proportions of dental professionals who obtain written consent ranging from 11 to 63.7% among the participants studied [21, 22, 24, 26–28]. The informed consent practices may be influenced by cultural contexts, differing legal standards for informed consent disclosure, and the working environment, including patient-dentist-related factors [1, 2, 29].

For Uganda, there is limited data on the informed consent process in dentistry with one available study reporting on the consent process among dental practitioners at the national referral hospital [30]. Thus, the objective of the present study was to determine the dentist’s perspectives, practices, and factors associated with the informed consent process for fixed prosthodontic treatment in Kampala Metropolitan area, Uganda. The null hypothesis was that dentists in Kampala Metropolitan area have good informed consent practices and there is no relationship between the consent practices and independent variables.

## Methods

### **Study design and approach**

This was a cross-sectional study carried out from August to September 2023 using quantitative and qualitative methods.

### Study setting and population

The study was conducted in Kampala metropolitan area. Kampala Metropolitan constitutes Kampala City, the capital of Uganda, and the surrounding districts of Wakiso, Mukono, and Mpigi. It is located in the central region of Uganda, which is the hub of dental services in the country [31]. According to available data on the dental workforce in Uganda, about 80% of dental surgeons work in urban areas, with Kampala, the capital city having the majority [31, 32]. The study population consisted of all dentists licensed to practice by the Uganda Medical and Dental Practitioners Council (UMPC) working within the study area.

### Sample size calculation and participant selection

The sample size was calculated using sample size formulae with a finite population correction:

$$n=\frac{{n}_{0}N}{{n}_{0}+(N-1)}$$,

where *n* was sample size; *n*_o_ = (Z^2^PQ)/d^2^; *N* was population size; *Z* is 1.96 (standard normal deviation at 95% confidence interval); *P* was the proportion (we arbitrarily used 50% as no previous study had been done in Africa); *Q* is 1 – *P*, *Q* = 1–0.5, therefore, *Q* = 0.5; and *d* was maximum error we allowed, *d* = 5% (95% confidence interval). The minimum sample size was estimated at 139 which was increased by 10% to 153 to accommodate for possible missing data.

The participants were selected based on lists of licensed dentists within Kampala Metropolitan from UMDPC. A total of 212 dentists were in active clinical practice. Using a simple random sampling technique, 153 dentists were selected for the main study. One dentist declined to participate and was excluded from the study as he was not active in clinical practice.

### Data collection methods

A semi-structured self-administered questionnaire which included both closed - and open-ended items in English was used to collect data. The questionnaire was adopted from two similar studies [24, 33] with some modifications. It comprised three sections. section I, solicited information on participants’ sociodemographic and service-related factors; whereas section II comprised perspectives-related questions including two items to assess awareness regarding the informed consent process. Perspectives toward the informed consent process were recorded using 10 items based on the 5-point Likert scale and 6 open-ended items. The items based on a Likert scale had alternatives: (1) “Strongly disagree”, (2) “Disagree”, (3) “Neutral”, (4) “Agree”, and (5) “Strongly Agree”. Participant responses were recorded by selecting the most appropriate answer from the hard copy questionnaire. Section III contained structured and open-ended items; 8 questions regarding the practices of dentists and items to capture challenges encountered or recommendations for improvement of the consent process. The questionnaire is attached as supplementary file 1.

During the main survey, two trained research assistants on scheduled appointments, visited the selected dentists in their dental facilities to administer informed consent and deliver a hard copy of the questionnaire. The participants were requested to fill out the questionnaire at a time convenient to them. On agreed dates, the research assistants returned to the dental facilities to collect completed questionnaires. A 98% (98.1%) response rate was achieved after two to three follow-up visits.

### Quality control

Before the commencement of the study, the questionnaire was pretested among 5 dentists to obtain feedback regarding the overall acceptability of the questionnaire in terms of language clarity and content coverage. The questionnaire was then pilot-tested among a convenient sample of 15 dentists working in Kampala who were excluded from the main survey. Feedback was obtained on the overall acceptability of the questionnaire in terms of length, language clarity, and to test for reliability and internal consistency. The items for perspectives and practices had a Cronbach’s alpha test result of 0.73. 14 out of the 15 ( 93.3%) participants found the questionnaire easy to complete. Based on the feedback received in the pilot study, a few modifications were made, which included adding multiple response variables for the type of consent used by the dentists.

The Principal Investigator trained two research assistants, who were dentists, regarding the study’s aim, and clarification of instruments. The completed questionnaire was checked for errors and completeness by the research assistants on collection. Double data entry into Epi data, version 3.1 was used to check for any errors and ensure completeness of the final data set.

### Variables

Dependent Variable: Informed consent practices.

Independent Variables: Socio-demographic characteristics (age, sex, highest education attained, work experience), Service-related factors (informed consent training, workload (number of patients treated per day), and the amount of time taken for obtaining consent).

### Statistical analyses

The data were imported from Epi data into STATA, version 14.0 (College Station TX, USA) for analysis. Correctly answered participant responses regarding perspectives were regarded as favourable perspectives. The responses for the ten items regarding perspectives were recorded to ensure that a high score indicated a favorable answer while a low score indicated a less favorable perspective. The overall and mean score for perspectives was computed. The responses were recategorized as disagree (1) for original categories strongly disagree to disagree, neutral (2), and agree (3) for strongly agree to agree. Descriptive statistics were used to summarize the data. Bivariate analysis was carried out to determine the presence of a significant relationship between each independent variable (socio-demographic and service-related factors) with obtaining written informed consent using generalized linear models using Poisson regression. Variables with a *p*-value of less than 0.20 at a 95% confidence interval in the binary poisson regression analysis were considered for the multivariable regression model. Backward elimination was used to drop the two most insignificant predictors (sex, type of practice) from the model; one at a time. The final model had four predictors considering a sample size of 153. Interaction and confounding were assessed; there was neither interaction nor confounding. Variables with *p*-value < 0.05 from the multivariate analysis were considered as having a statistically significant association with the practice of obtaining written informed consent. The multivariate model was evaluated by using the goodness of fit method to check whether the model fitted the data well and obtained a *p*-value of greater than 5%.

Responses to open-ended items were analyzed using content and thematic analysis using a combination of deductive and inductive approaches and interpreted in line with the study objectives and research questions. In content analysis, the explicit words or phrases were coded for the existence of a concept. Thereafter, the codes were grouped into meaningful sets of sub-categories and categories. In addition, some of the coded qualitative data were summarised numerically with descriptive statistics, and proportions of participants’ texts with particular codes were computed.

## Results

### Socio-demographic and service-related factors of participants

Most (86.9%) of the participants were general dentists. Nearly two-thirds were male (64.7%) and 57.0% had experience of more than five years of dental practice. The majority (75.1%) of the participants worked in private dental facilities. All participants provided at least one fixed prosthodontic prosthesis in the last month (Table 1). Questions about age or average number of patients treated with fixed prostheses were not answered by three or five participants, respectively.

**Table 1:** Frequency distribution of participants according to demographic factors (*n* = 153 )

Characteristic	Category	Freq (*n*)	Perc (%)
**Socio-demographic factors**			
Cadre	General dentists	133	86.9
	Specialists	20	13.1
Type of dental practice	Government aided-facility	33	21.6
	Private dental practice	115	75.1
	PNPF	5	3.3
Sex	Female	54	35.3
	Male	99	64.7
Age category (in years, *n* = 148)	24 to 35	84	56.8
	36 to 45	48	32.4
	46 to 60	16	10.8
Work of experience	< 5	52	34.0
(in years)	5–10	44	28.8
	> 10	57	37.2
**Service-related factors**			
Training in informed consent	Yes	77	50.3
Average number of patients treated per day (*n* = 150 )	< 5	41	27.3
	5–9	74	49.3
	10 and above	35	23.4
Average time spent on obtaining consent (in minutes)	Upto 5	32	20.9
	6–15	45	29.4
	> 15	76	49.7
Types of fixed prosthesis delivered in the last month	Crown	149	97.4
	Bridge	109	71.2
	Inlay	21	13.7
	Onlay	15	9.8

*PNFP-Private Not For Profit, * FPT- Fixed Prosthodontic Treatment

### Perspectives awareness of dentists about the informed consent process

The dentists had a good understanding of what informed consent is, as they defined the term well with all its elements. For example, two participants defined informed consent as:

“*Giving a patient all the relevant information about the prescribed treatment/treatment plan and making sure the patient has understood the information given that includes advantages, disadvantages, and risks, based on the information provided the patient makes a voluntary decision about the treatment*”.

Another participant defined consent as:

*A process in which a patient is given important information about a procedure or treatment including possible risks and benefits and makes an informed decision based on the information given*.

Overall, the dentists’ perspectives regarding application consent for FPT were favorable. Almost all of the participants (94.8%) agreed that a dentist should obtain informed consent before FPT and 92.2% agreed that written informed consent is essential for invasive dental treatment procedures for example FPT (Table 2). Furthermore, 98.7% agreed that patients had a right to participate in decisions regarding their treatment.

Participants had favorable perspectives about the legal purpose of informed consent as more than three-quarters (> 75.0%) of the participants had favorable views with statements numbered Q3 to Q5 (Table 2). Furthermore, the participants held favorable perspectives about the provision of information and the necessity to understand (Q7 to Q9), however, a considerable percentage (32.0%) of them thought that there is limited time in daily practice for consent (Table 2). In addition, the majority (92.8%) felt that the use of additional educational materials like brochures, and videos describing a procedure may facilitate the consent process for FPT.

**Table 2:** Perspectives of dentists regarding informed consent to fixed prosthodontic treatment

No.	Statement	Agree *n*(%)	Neutral *n*(%)	Disagree *n*(%)
Q1	Dentists should obtain informed consent before FPT	145(94.8)	1(0.6)	7(4.6)
Q2	Written informed consent is essential for all invasive dental procedures for example FPT	141(92.2)	8(5.2)	4(2.6)
Q3	Written informed consent is a protective shield for the dentist and dental practice	137(89.6)	8(5.2)	8(5.2)
Q4	A consent form is meant to protect the patient’s right	116(75.8)	23(15.0)	14(9.2)
Q5	Signing the consent form is just a formality	12(7.8)	11(7.2)	130(85.0)
Q6	Signing a consent form proves that the patient understood the nature of the procedure and the consequence(s) of the FPT	129(84.3)	9(5.9)	15(9.8)
Q7	Dentists should at least describe to the patient the nature of the procedure, benefits, risks, and any alternative treatments of the FPT	143(93.5)	3(1.9)	7(4.6)
Q8	There is a need to consider the wishes of the patient and the family regarding the amount of information they need to know	109(71.2)	20(13.1)	24(15.7)
Q9	Every effort should be made to explain all the facts regarding the FPT to the patient in simple language they can understand	146(95.4)	1(0.7)	6(3.9)
Q10	There is limited time for obtaining truly informed consent for FPT in daily clinical practice	49(32.0)	21(13.7)	83(54.3)

### Practices of informed consent for FPT

**Fig. 1:**
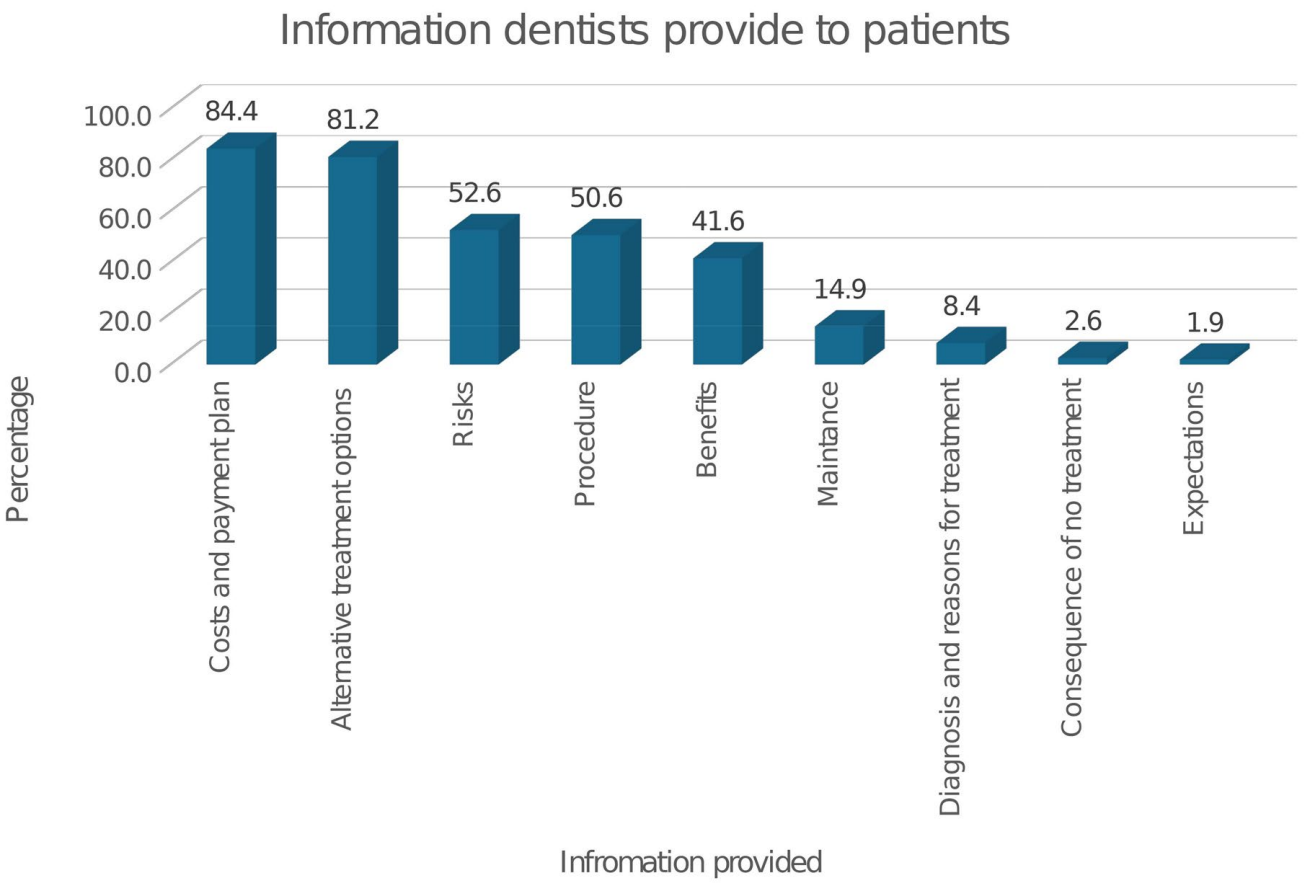
Information provided by dentists during the consent process for fixed prosthodontic treatment

Overall, there were some variations in the dentists’ practices regarding informed consent for FPT. Most of the participants reported that they always obtain informed consent for FPT and use oral consent, 87.6%, and 81.6% respectively. About one-third (29.4%, *n* = 45) of the dentists reported using written consent for fixed prosthodontic treatment with some of them using both oral and written consent depending on the case (Table 3). Most of the dentists reported providing information regarding the costs for treatment and alternative treatment options including their advantages and disadvantages (Fig. 1). About half of the study participants reported providing information regarding the treatment procedure, risks, or benefits (Fig. 1).

**Table 3:** Informed consent practices for FPT among dentists in Kampala metropolitan area

Practice	Frequency	Percentage (%)
**Obtain informed consent**		
Yes always	134	87.6
Sometimes	11	7.2
No	8	5.2
**Type of informed consent used (multiple responses)**		
Written consent	45	29.4
Oral consent	124	81.6
Implied or presumed	12	7.9
I don’t take informed consent	2	1.3
Both oral and written consent	23	15.0
**Cadre who obtains consent**		
Dentist who will treat the patient	143	93.5
Junior dentists	1	0.6
Nurses/Chair side assistant	7	4.6
Receptionist	2	1.3
**Patients from whom the dentist may not obtain consent**		
Colleague	15	9.8
Relative /friend	14	9.2
Long-time patient	5	3.3
None of them	118	77.1
other	1	0.6

### Factors associated with obtaining written informed consent

Bivariate Poisson regression analysis revealed that having more than 10 years of working experience and having had training on informed consent during service had a statistically significant positive association with obtaining written informed consent practice. With multivariate analysis, having more than 10 years of working experience had a statistically significant positive association with obtaining written informed consent practice (Table 4).

**Table 4:** Association of independent variables and the practice of obtaining written consent

Variable	Obtaining written consent		Bivariate analysis		Multivariate analysis	
	Yes *n*(%)	No *n*(%)	PR	*p*-value (confidence interval)	Adjusted PR	*p*-value (confidence interval)
**Cadre**						
General Dentist	36(27.1)	97(72.3)	1			
Specialist	9(45.0)	11(55.0)	1.6625	0.076(0.9488–2.9231)	-	-
**Type of practice**						
Government	6(18.2)	27(81.8)	1			
Private/PNFP	81(67.5)	39(32.5)	1.7875	0.140 (0.8269–3.8636)	2.0332	0.065 (0.9561–4.323)
**Age (in years)**						
24 to 35	23(27.4)	61(72.6)	1			
36 to 45	16(33.3)	32(66.7)	1.2174	0.469 (0.71497–2.0729)	-	-
45 to 60	5(32.3)	11(68.7)	1.1413	0.749 (0.5084–2.5621)	-	-
**Sex**						
Female	11(20.4)	43(79.6)	1	1	-	-
Male	34(34.4)	65(65.6)	1.6840	0.086 (0.9295–3.0581)	-	-
**Years of experience**						
upto 5	9(17.3)	43(83.7)	1	1	1	1
6 to 10	14(31.8)	30(68.2)	1.838	0.106 (0.8795–3.843)	1.7729	0.125 (0.8526–3.6865)
Above 10	22(38.6)	35(61.4)	2.230	0.021 (1.1291–4.4043)	2.1474	0.030 (1.0782–4.2771)
**Training on informed consent**						
Yes	29(37.7)	48(62.3)				
No	16(21.1)	60(78.9)	0.5590	0.029 (0.3312–0.9433)	0.6279	0.088 (0.3671–1.0723)
**Duration of obtaining consent process**						
(in minutes) < 5	4(36.6)	7(63.6)	1	1	-	-
5 to 15	19(28.8)	47(71.2)	0.7917	0.599 (0.3311–1.8930)	-	-
> 15	22(28.9)	54(71.1)	0.7961	0.603 (0.3368–1.8817)	-	-

*PR-prevalence ratio; 1*-was taken as reference; *p* -value < 0.05 was considered significant; Poisson regression was used for analysis

In response to the open-ended questions, the most frequent challenges reported were under the category of language barriers and communication. These challenges included difficulty in communicating technical information, language barriers, and misunderstandings or misconceptions about treatment information. Other issues included patients’ preference for dentists to make decisions, contradicting decisions made by patients and their support teams, and reluctance or misconceptions with consent form signing. The recommendations included training about informed consent, streamlining guidelines for informed consent in dentistry for Uganda, and the development of standardized consent forms.

In response to open-ended questions, the most frequent challenges and recommendations were broadly categorized as;

Challenges

- Language barrier and communication that included difficulty in communicating technical information, language barriers, and misunderstandings or misconceptions about treatment information.
- Challenges in decision-making that included patients’ preference for dentists to make decisions, contradicting decisions made by patients and their support teams,
- Reluctance or misconceptions with signing consent forms.

Recommendations

- Training about informed consent,
- Streamlining guidelines for informed consent in dentistry for Uganda.
- Development of standardized consent forms.

## Discussion

The present study provides baseline information that provides a better understanding of the dentist’s perspectives and practices regarding informed consent for fixed prosthodontic treatment. The findings indicate that the majority of the dentists were familiar with the term and elements of informed consent and held favorable perspectives toward it (Table 2). However, despite the largely positive disposition towards consenting and knowledge of its importance in FPT, overall, dentists’ practices were inadequate as less than a third reported obtaining written consent (Table 3), and about half did not disclose the risks, planned procedures, or benefits of the treatment (Fig. 1). Thus, the informed consent practice is adequate and still looks as it is theoretical ideal among dentists in Kampala, Uganda. This undermines the goals of informed consent to protect patient rights and guide ethical clinical practice. Thus, patients may have to undergo fixed prosthodontics without having sufficient knowledge or understanding of treatments.

This study’s finding regarding dentists’ familiarity with informed consent and a majority having favorable perspectives, concurs with literature from various regions of the world, including Bulgaria, India, and Pakistan [22–24, 26, 34]. In India, Gupta and colleagues found that the majority (91%) of dentists had a good awareness of informed consent, and 89.7% of them felt it was necessary to explain the treatment plan before obtaining consent [22]. In Bulgaria, Avramova and Yaneva found that 97.5% of the dentists felt that informed consent was necessary [24]. In contrast, a study among surgeons at three tertiary hospitals in Uganda found that most surgeons could not define the term “informed consent” [33]. The difference could be due to the difference in the study population.

It is noteworthy to mention that most dentists indicated that signing a consent form proves that the patient understood the nature of the procedure and the consequence(s) of the FPT (Q6, Table 2). However, they also reported that misunderstandings or misconceptions of treatment information as one of the most frequent challenges. Literature points out that merely signing the form does not necessarily prove the patient understood the information provided unless an additional comprehension test supports that claim [5, 29, 35]. Remarkably, poor patient communication, unmet patient expectations, and a failure to obtain informed consent were reported as potential sources of malpractice by Nassani on reviewing literature about prosthodontic malpractice [10]. These could be a consequence of misconceptions, and misunderstanding of treatment-related information or provision of inadequate information to patients. Thus it has been suggested that dentists can potentially reduce the possibility of malpractice claims and better manage patient expectations by making sure patients completely understand the treatment process, risks, and alternatives through a thorough informed consent process [10]. These study findings may suggest a need for education on consent, as well as the creation of innovative ways and tools that will facilitate comprehension.

In the present study, the results reveal that dentists predominantly used oral consent compared to written consent at (81.6%) against (29.1%) respectively (Table 2). This finding is similar to results observed in Brazil, Pakistan, India, and Bulgaria [15, 24–26, 28]. A study from Brazil found that only 14.5% of dentists reported using informed consent forms every time; 48%, sometimes, and the rest only in special cases [15]. In Bulgaria, 46.3% of dentists obtained verbal consent; 37.5%, written informed consent, and 16.25% used both forms for dental care.

Variations in the type of consent used for fixed prosthodontics may result from a variety of factors, including competing healthcare demands, time, or the differing legal standards for informed consent in different countries or strictness of enforcement [2, 35, 36]. For example, in Uganda, the guidelines regarding informed are not specific; the patient’s charter broadly states that consent may be verbal, implied, or written with no specific guidelines for different dental procedures [18]. Similarly, the professional code of ethics by the UMDPC only states that “a practitioner shall not conduct any intervention or treatment without consent except where a bonafide emergency obtains” in section 7b [17]. Without detailed guidelines, there may be variations in the practices of dentists.

The present study finding that 29.4% of the dentists obtain written consent, differs from the results of the previous Ugandan study by Nono and colleagues who reported that only 5.3% of dental practitioners used written consent at a national government facility [30]. This discrepancy may be due to the extensive study area coverage for the present study, the difference in study participants, and the specific procedure studied. In the present study, dentists recruited work in different types of dental facilities which may vary in nature of patient flow, clinicians’ experience, and standards of dental facilities as compared to the study conducted in a single government institution. In addition, the present study reports on informed consent practices for fixed prosthodontic treatment, an invasive dental procedure.

The present findings showed variation in the information provided by the dentists during the consent process. Most dentists disclosed information regarding payment and alternative treatments while about half of dentists provided information regarding the procedure, benefits of the chosen treatment, and risks (Fig. 1). Globally, available literature from other medical specialties reports similar results as there are gross variations in the information given before consent to different medical treatments [37–42]. In a tertiary care hospital in India, Bhushan and Manhas noted that although the majority of the patients were informed about the procedure, indication, benefits, and risks, very few were aware of their options for alternative procedures and the right to refuse the procedure before cesarean Sect. [37]. In Ethiopia, Chane and colleagues also, reported that only 8.1% of the patients who underwent surgery had received the minimum required components of informed consent that included type of the surgery, benefits, risks of the procedure, and alternative options of treatment [38]. Dentists should communicate all the information needed for patients to make informed decisions regarding their treatment which may help to better manage patient expectations and potentially reduce the likelihood of malpractice claims [10]. According to several authors, clinical consent information must contain four components that include: information about the description of the procedure, anticipated risks, benefits, and available alternatives [4, 5, 39]. Most dentists disclosed information about payment and alternative treatments, probably because they thought that this was the most crucial information patients needed to know in order to make decisions regarding their treatment.

In the present study, having had a working experience was the only factor significantly associated with obtaining written informed consent. In contrast, Negash and colleagues found several factors including age of above 35 years, more than 10 years of working experience, having training on informed consent, and spending more time (> 30 min) to obtain consent were significantly associated with good informed consent practice. The differences in findings with the present study may be due to methods of analysis and the smaller sample size of the present study [36].

### Study strengths and limitations

The present study provided the baseline information necessary to understand the perspectives and practices regarding informed consent for fixed prosthodontic treatment among dentists in Kampala Metropolitan area, Uganda. The study limitations included; (1) The assessment of the perspectives and practices was based on dentists’ self-reports; thus we could not rule out response bias, however, several open-ended questions were used in an attempt to reduce such bias. (2) The findings of the present study may not be generalized to all dentists in Uganda as participants were recruited from one region of Uganda.

### Conclusion and recommendations

While most dentists were familiar with the concepts of informed consent and had favorable perspectives towards it, their actual practices were inadequate. In conclusion, informed consent practice still looks as if it is a theoretical ideal. Addressing the identified gaps in the informed consent process may lead to better protection of patient rights and improvement in the quality of dental care services in Kampala, Uganda. Therefore, it is recommended that periodic training courses in informed consent process in dentistry be designed, as well as the development of standardized consent forms specifically tailored to fixed prosthodontic treatment as strategies to improve current practices and ensure consistency in the information provided. In order to provide a more holistic picture regarding the informed consent process, future research should identify the barriers to obtaining written consent as well as studies to explore patient experiences, comprehension information provided, and studies to include a broader and more diverse sample of dentists across Uganda. Furthermore, research is required to streamline guidelines for the informed consent process in dental care in Uganda.

### Clinical significance of the study

The present study provided the baseline information necessary to understand the perspectives and practices regarding the informed consent process for fixed prosthodontic treatment among dentists in the Kampala Metropolitan area, Uganda. The findings may inform the design of future studies and strategies to improve the consent process for fixed prosthodontic treatment and other dental procedures in Uganda and other low-income countries.

### Electronic supplementary material

Below is the link to the electronic supplementary material.

Supplementary Material 1

Supplementary Material 2

## Data Availability

Data sources are available on request. The request can be sent to the corresponding author at barbarandagire@yahoo.com.
