# Supplementary material for: Dentists’ perspectives, practices, and factors associated with informed consent process for fixed prosthodontic treatment: a cross-sectional study of kampala metropolitan area, Uganda
Source: BMC Oral Health. 2024 May 27;24:614. doi: 10.1186/s12903-024-04380-w (PMC11131203; doi:10.1186/s12903-024-04380-w)
Supplement: Supplementary file 1 — Supplementary Material 1 [file 12903_2024_4380_MOESM1_ESM.docx]

**Questionnaire for Dentists**

**Title of Sub-Study: Dentists Perspectives and Practices Regarding Informed Consent for Fixed Prosthodontic Treatment in Kampala Metropolitan Area, Uganda**

1. **Study number .........................................................................................................**

**Section I: Socio-Demographic Data**

1. Sex: ..........................................................................................................................
2. Age: ..........................................................................................................................
3. Highest level of education attained: .........................................................................
4. Please select the option that applies
5. Intern dentist
6. General dental practitioner
7. Specialist, please specify…………………………………………………..
8. Please mention the number of years in clinical practice: ...........................................
9. Type of dental practice in which you mainly work
10. Government Facility
11. Private Not-For-Profit Organisation
12. Private Dental Practice
13. Location of the dental practice in which you mainly work
14. Kampala district
15. Wakiso district
16. Mukono district
17. Mpigi district
18. Have you received training on informed consent after completing your university studies?
19. Yes
20. No
21. Average number of dental patients you treat in one day:……………………………
22. Average number of patients you see in a month whom you treat with fixed prostheses………………………………………
23. Please select/circle the type(s) of fixed dental prosthesis(es) you have delivered in the last one year
24. Bridge, b. Crown, c. Inlay,

d. Onlay e. other, please specify:……………………….

**Section II: Perspectives Regarding Informed Consent**

1. Briefly explain what you understand by the term informed consent ……………………………………………………………………………………………………………………………………………………………………………………………………………………………………………………………………………………………………………………………………………………………………………………………………………………………………………………………………
2. Do you think that it is necessary to obtain informed consent from all dental patients?

- Yes
- No
  - If no, please explain………………………………………………………………………….
- Do not know

1. Is it necessary to obtain informed consent from patients for fixed prosthodontic treatment?
2. Yes
3. No
4. If no, please explain………………………………………………………………………….
5. Do not know
6. When should a dentist obtain informed consent for patients who need fixed prosthodontic treatment?

……………………………………............................................................................................................................................................................................................................

1. Who should obtain consent from patients for their fixed prosthodontic dental treatment?

……………………………………………………………………………………………………………………………………………………………………………………………………………………………………………………………………………………………………………………………………………………………………........

1. What information should be provided to the patients before obtaining consent for fixed prosthodontic treatment? ........................................................................................................................................................................................................................................................................................................................................................................................................................................................................................................................................................................................................................................................................................................................................................................................................................................................................................................................................................................................
2. Do patients have a right to participate in treatment decisions of management with a fixed dental prosthesis?
3. Yes
4. No
5. If no why not? ......................................................................................................................................

**Please choose the most appropriate response regarding each of the following statements by placing a tick in the appropriate box**

| **No.** | **Statement Regarding Consent** | **Strongly Disagree** | **Disagree** | **Neutral** | **Agree** | **Strongly Agree** |
| --- | --- | --- | --- | --- | --- | --- |
| 20 | Dentists should obtain Informed consent before fixed prosthodontic treatment |  |  |  |  |  |
| 21 | A consent form is meant to protect patient rights |  |  |  |  |  |
| 22 | Signing the consent form proves that the patient understood the nature of the procedure and the consequence(s) of the fixed prosthodontic treatment |  |  |  |  |  |
| 23 | Signing the consent form is just a formality |  |  |  |  |  |
| 24 | Written informed consent is a protective shield for the dentist and dental practice |  |  |  |  |  |
| 25 | Written informed consent is essential for all invasive dental procedures for example fixed prosthodontic treatment |  |  |  |  |  |
| 26 | The dentist should at the very least describe to the patient the nature of the procedure, benefits, risks, and any alternate treatments of the fixed prosthodontic treatment. |  |  |  |  |  |
| 27 | There is a need to consider the wishes of the patient and the family regarding the amount of information they need to know. |  |  |  |  |  |
| 28 | In daily dental practice, there is limited time for obtaining truly informed consent for fixed prosthodontic treatment |  |  |  |  |  |
| 29 | Every effort should be made to explain all the facts regarding fixed prosthodontic treatment to the patient in simple language they can understand |  |  |  |  |  |
| 30 | Educational materials like brochures, and videos, describing a procedure may facilitate the consent process for fixed prosthodontic treatment. |  |  |  |  |  |

**Section III: Practices Regarding Informed Consent for Fixed Prosthodontic Treatment**

31. Do you obtain informed consent from your patients?

- Yes, always
- No
- Only sometimes for certain cases, please give examples ……………………………………………………………………………………………………………………………………………………………………………………

………………………………………………………………………………………….

1. What form of informed consent do you obtain in your practice? Please select the option(s) that apply

- I don’t take informed consent
- Implied
- Oral
- Written
- Do not know

1. What form of informed consent do you obtain for fixed prosthodontic treatment? Please select the option(s) that apply

- I don’t take informed consent
- Implied or presumed
- Oral
- Written
- Do not know

1. If you take informed consent, how long does the consenting procedure typically take for patients who need fixed prosthodontic treatment?................................................
2. Who obtains informed consent from patients who seek fixed prosthodontic treatment in your practice or dental clinic?

- Receptionist
- Nurses/ Chair-side assistant
- Junior dentists
- Dentist who will treat the patient
- Other, please specify …………………………………………………………………………………………

1. Are there any patients who seek fixed prosthodontic treatment from whom you might not wish to obtain informed consent?

- Colleague
- Relative friend
- Long-time patient
- None of them

1. Do you provide information to patients before management with a fixed prosthodontic treatment?

- Yes, always
- No
- Sometimes for certain cases. Please give examples ……………………………………………………………………………………………………………………………………………………………………………………

1. If you do give information to patients, what information do you provide to patients before management with a fixed prosthodontic treatment? ........................................................................................................................................................................................................................................................................................................................................................................................................................................................................................................................................................................................................................................................................................................
2. Have you experienced any challenges when obtaining consent for fixed prosthodontic treatment?

- Yes
- No
- If yes, please specify ……………………………………………………………………………………………............................................................................................................................................................................................................................................................................

1. Do have any other comments regarding informed consent for fixed prosthodontic treatment? .......................................................................................................................................

………………………………………………………………………………………………………………………………………………………………………………………………………………………………………………………………………………

**Thank you for your assistance. It is much appreciated!**
