# Supplementary material for: Dentists’ perspectives, practices, and factors associated with informed consent process for fixed prosthodontic treatment: a cross-sectional study of kampala metropolitan area, Uganda
Source: BMC Oral Health. 2024 May 27;24:614. doi: 10.1186/s12903-024-04380-w (PMC11131203; doi:10.1186/s12903-024-04380-w)
Supplement: Supplementary file 2 — Supplementary Material 2 [file 12903_2024_4380_MOESM2_ESM.doc]

STROBE Statement—Checklist of items that should be included in reports of ***cross-sectional studies***

|  | Item No | Recommendation | Reported on Page Number |
| --- | --- | --- | --- |
| **Title and abstract** | 1 | (*a*) Indicate the study’s design with a commonly used term in the title or the abstract  Cross-sectional study as stated in the title on page 1, and abstract page 2 | Pg 1  Pg 2 |
|  |  | (*b*) Provide in the abstract an informative and balanced summary of what was done and what was found  Provided in abstract page 2 | Pg 2 |
| Introduction | | |  |
| Background/rationale | 2 | Explain the scientific background and rationale for the investigation being reported  Included in the introduction pages 3 and 4 | Pg 3,4 |
| Objectives | 3 | State specific objectives, including any prespecified hypotheses  Included in the introduction page 5 | Pg 5 |
| Methods | | |  |
| Study design | 4 | Present key elements of study design early in the paper  Included in the methods page 5 | Pg 5 |
| Setting | 5 | Describe the setting,  locations, and  relevant dates,  including periods of recruitment,  exposure, follow-up, and data collection  Included in the methods on pages 5 and 6 | Pg 6  Pg 7 |
| Participants | 6 | (*a*) Give the eligibility criteria,  and the sources and methods of selection of participants  Included in the method page 6 | Pg 6 |
| Variables | 7 | Clearly define all outcomes, exposures, predictors, potential confounders, and effect modifiers. Give diagnostic criteria, if applicable  Include in methods page 8 | Pg 8 |
| Data sources/ measurement | 8* | For each variable of interest, give sources of data and details of methods of assessment (measurement). Describe comparability of assessment methods if there is more than one group  Included in methods page 7 | Pg 7 |
| Bias | 9 | Describe any efforts to address potential sources of bias  Included in the quality control page 7 and the study strengths and limitation page 22 | Pg 7  Page 22 |
| Study size | 10 | Explain how the study size was arrived at  Includes in sample size calculation page 6 | Pg 6 |
| Quantitative variables | 11 | Explain how quantitative variables were handled in the analyses. If applicable, describe which groupings were chosen and why  Included methods page 9 | Pg 9 |
| Statistical methods | 12 | (*a*) Describe all statistical methods, including those used to control for confounding  Included in the methods page 9 | Pg 9 |
|  |  | (*b*) Describe any methods used to examine subgroups and interactions |  |
|  |  | (*c*) Explain how missing data were addressed  Included on page 7 and page 10 | Pg 7 and 10, |
|  |  | (*d*) If applicable, describe analytical methods taking account of sampling strategy  Included in methods page 9 | Pg 9 |
|  |  | (*e*) Describe any sensitivity analyses  Not applicable |  |
| Results | | |  |
| Participants | 13* | (a) Report numbers of individuals at each stage of study—eg numbers potentially eligible, examined for eligibility, confirmed eligible, included in the study, completing follow-up, and analysed  Included page 10 | Pg 10 |
|  |  | (b) Give reasons for non-participation at each stage  Included methods page 6 and results page 10 | Pg 6 and 10 |
|  |  | (c) Consider use of a flow diagram  This is not required |  |
| Descriptive data | 14* | (a) Give characteristics of study participants (eg demographic, clinical, social) and information on exposures and potential confounders  Included in results page 10-13; Summarized table 1, 2 | Pg 10, 13 |
|  |  | (b) Indicate number of participants with missing data for each variable of interest  Included in the results page 10 | Page 10 |
| Outcome data | 15* | Report numbers of outcome events or summary measures  Include in results page 14 to 16, summarised in Table 3, 4 an ffigure 1 | Pg 11 to 16 |
| Main results | 16 | (*a*) Give unadjusted estimates and, if applicable, confounder-adjusted estimates and their precision (eg, 95% confidence interval). Make clear which confounders were adjusted for and why they were included  Included in the results page 16 | Pg 15 |
|  |  | (*b*) Report category boundaries when continuous variables were categorized  Included in results and abstract pages 2, 10, 16; summarized in table 1, 4 | Pg 2, 10 and 16 |
|  |  | (*c*) If relevant, consider translating estimates of relative risk into absolute risk for a meaningful time period  Not Applicable |  |
| Other analyses | 17 | Report other analyses done—eg analyses of subgroups and interactions, and sensitivity analyses  Not Applicable |  |
| Discussion | | |  |
| Key results | 18 | Summarise key results with reference to study objectives  Included in the discussion page 17 | Pg 17 |
| Limitations | 19 | Discuss limitations of the study, taking into account sources of potential bias or imprecision. Discuss both direction and magnitude of any potential bias  Included in the limitations page 22 | Pg 22 |
| Interpretation | 20 | Give a cautious overall interpretation of results considering objectives, limitations, multiplicity of analyses, results from similar studies, and other relevant evidence  Included in the discussion page 17-22 | Pg 17-22 |
| Generalisability | 21 | Discuss the generalisability (external validity) of the study results  Included in the limitation page 22 | Pg 22 |
| Other information | | |  |
| Funding | 22 | Give the source of funding and the role of the funders for the present study and, if applicable, for the original study on which the present article is based  Included page 23 | Pg 23 |
